# DNA repair and crossing over favor similar chromosome regions as discovered in radiation hybrid of *Triticum*

**DOI:** 10.1186/1471-2164-13-339

**Published:** 2012-07-24

**Authors:** Ajay Kumar, Filippo M Bassi, Etienne Paux, Omar Al-Azzam, Monika Michalak de Jimenez, Anne M Denton, Yong Q Gu, Eric Huttner, Andrzej Kilian, Sachin Kumar, Aakash Goyal, Muhammad J Iqbal, Vijay K Tiwari, Munevver Dogramaci, Harindra S Balyan, Harcharan S Dhaliwal, Pushpendra K Gupta, Gursharn S Randhawa, Catherine Feuillet, Wojciech P Pawlowski, Shahryar F Kianian

**Affiliations:** 1 Department of Plant Sciences, North Dakota State University, Fargo, ND, 58102, USA; 2 INRA-UBP 1095, Genetics Diversity and Ecophysiology of Cereals, Clermont-Ferrand, 63100, France; 3 Department of Computer Science, North Dakota State University, Fargo, ND, 58102, USA; 4 USDA-ARS, Western Regional Research Center, Albany, CA, 94710, USA; 5 Diversity Arrays Technology Pty Ltd, Yarralumla, ACT, 2600, Australia; 6 Department of Genetics and Plant Breeding, Ch. Charan Singh University, Meerut, 25004, India; 7 Department of Crop and Soil Science, Oregon State University, Corvallis, OR, 97331, USA; 8 USDA-ARS, Biosciences Research Laboratory, Fargo, ND, 58102, USA; 9 Akal School of Biotechnology, Eternal University, Baru Sahib, 173101, India; 10 Department of Biotechnology, Indian Institute of Technology, Roorkee, 247667, India; 11 Department of Plant Breeding and Genetics, Cornell University, Ithaca, NY, 14853, USA

**Keywords:** Non homologous end joining, Physical mapping, Gamma radiation, Deletion mutant, Chromatin, Wheat chromosome 3B, Radiation hybrid

## Abstract

**Background:**

The uneven distribution of recombination across the length of chromosomes results in inaccurate estimates of genetic to physical distances. In wheat (*Triticum aestivum* L.) chromosome 3B, it has been estimated that 90% of the cross over events occur in distal sub-telomeric regions representing 40% of the chromosome. Radiation hybrid (RH) mapping which does not rely on recombination is a strategy to map genomes and has been widely employed in animal species and more recently in some plants. RH maps have been proposed to provide *i*) higher and *ii*) more uniform resolution than genetic maps, and *iii*) to be independent of the distribution patterns observed for meiotic recombination. An *in vivo* RH panel was generated for mapping chromosome 3B of wheat in an attempt to provide a complete scaffold for this ~1 Gb segment of the genome and compare the resolution to previous genetic maps.

**Results:**

A high density RH map with 541 marker loci anchored to chromosome 3B spanning a total distance of 1871.9 cR was generated. Detailed comparisons with a genetic map of similar quality confirmed that *i*) the overall resolution of the RH map was 10.5 fold higher and *ii*) six fold more uniform. A significant interaction (r = 0.879 at *p* = 0.01) was observed between the DNA repair mechanism and the distribution of crossing-over events. This observation could be explained by accepting the possibility that the DNA repair mechanism in somatic cells is affected by the chromatin state in a way similar to the effect that chromatin state has on recombination frequencies in gametic cells.

**Conclusions:**

The RH data presented here support for the first time *in vivo* the hypothesis of non-casual interaction between recombination hot-spots and DNA repair. Further, two major hypotheses are presented on how chromatin compactness could affect the DNA repair mechanism. Since the initial RH application 37 years ago, we were able to show for the first time that the *iii*) third hypothesis of RH mapping might not be entirely correct.

## Background

Genetic mapping has been the foundation of molecular analysis in plants and animals for nearly a century, since the publication of the first map by Sturtevant in 1913 [1]. This widely successful approach relies on recombination to separate and order marker loci. In many plant species, including wheat (*Triticum aestivum* L.), recombination events are not evenly distributed along the length of the chromosomes [2-7]. Recombination hot-spots, sites with high recombination rates, are interspersed with recombination cold-spots. In addition, in species with large genomes, such as wheat, barley, or maize, recombination frequency tends to decrease with increased proximity to the centromere [5,7], being close to zero at the centromere and its surroundings. It is estimated that about one-fourth to one-third of the ~17 Gb wheat genome [8] accounts for less than 1% of the total recombination [3,5,9]. Initial studies hypothesized that these recombination poor regions were nothing more than “junk” DNA [10,11], only to discover that over 30% of wheat genes exist within this space [5]. Limited recombination makes these genes virtually inaccessible to genetic mapping. Similarly, the use of genetic maps as scaffolds to orient physical maps (such as ordered BAC contigs) provides only limited information for these recombination poor regions.

Radiation hybrid (RH) mapping is a method that was originally proposed as an alternative to the use of recombination for mapping marker loci [12,13]. In RH mapping, high dosages of radiation are used to generate random double strand breaks (DSBs) across the genome. The DSBs are then recognized and fixed by one of two main repair mechanisms: homology-directed repair (HR) or non-homologus end-joining (NHEJ), also known as illegitimate recombination [14-17]. Both of these mechanisms are highly conserved in eukaryotes. NHEJ, an error prone mechanism, is considered the prevailing choice of somatic DSB repair in higher eukaryotes [16]. In the last two decades, a number of proteins involved in the NHEJ repair mechanism have been identified [15,16,18,19]. Also, a model has been proposed to explain their interaction and functionality. In brief, presence of DNA broken ends is sensed through the ATM (Ataxia Telangiectasia Mutated) signaling pathway. The *Ku* protein complex is then recruited to the damaged site with the function of protecting the DSBs from further degradation. The *Ku* complex becomes anchored at the break site and is then used as a docking point by DNA phosphokinases, which directly or indirectly create protein bridges to pull the two broken ends together, and finally re-join them by a DNA ligase [16,18,19]. When the broken ends are re-joined, the nucleotides located within the adjacent DSBs are lost. The loss mainly involves a small number of nucleotides, but deletions of larger size are not uncommon [14,16,17].

Radiation hybrid mapping exploits the formation of DNA deletions to generate a binary polymorphism (1-retention *vs.* 0-deletion) which is then used to identify the correct marker order by their simultaneous co-deletion or co-retention [12]. While the molecular components of the NHEJ mechanism of repair have been partially or entirely identified, its precise activity in live organisms requires further investigations. In this regard, viable RH plant populations might represent a novel and useful tool.

During the last two decades, RH mapping has played an important role in mapping and genome assembly of a number of organisms, including humans and other animals [20-29]. However, only a few preliminary studies have been reported in crop plants, such as maize (*Zea mays* L.) [30,31], barley (*Hordeum vulgare* L.) [32], cotton (*Gossypium hirsutum* L.) [33] and wheat [34-37]. In an effort to sequence the bread wheat chromosome 3B, a partial physical map covering 82% of the estimated 993 Mb size of the chromosome was recently published [36]. In that study, RH mapping was tested as a mean to provide good quality scaffolding for physical mapping. Here, we present an extension of that work, with the development of a high density RH map of chromosome 3B to align bacterial artificial chromosome (BAC) contigs. This is an unprecedented opportunity in plants to examine the physical distribution of radiation mediated deletions at a very refined level. Furthermore, this map was employed to assess what are commonly considered the three major advantages of RH mapping: *i*) high map resolution, *ii*) precise conversion of centi Rays (cR, map unit of RH) distances to physical distances, and *iii*) independence from the patterns observed for meiotic recombination events. Surprisingly, the data presented here support only the first two hypotheses, while a strong correlation was identified between the action of the DNA breakage/repair mechanism and the frequency of meiotic recombination events. This result suggests that the state of chromatin may influence the DNA break formation and repair mechanism in a similar way as it influences recombination events.

## Results

### A dense and precise 3B-RH map

**Figure 1:**
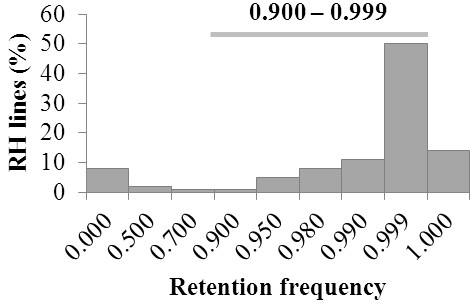
**Distribution of marker retention frequencies.** The frequencies were calculated from a population of 92 radiation hybrid lines specifically created for chromosome 3B of wheat and are based on 541 marker analysis.

**Figure 2:**
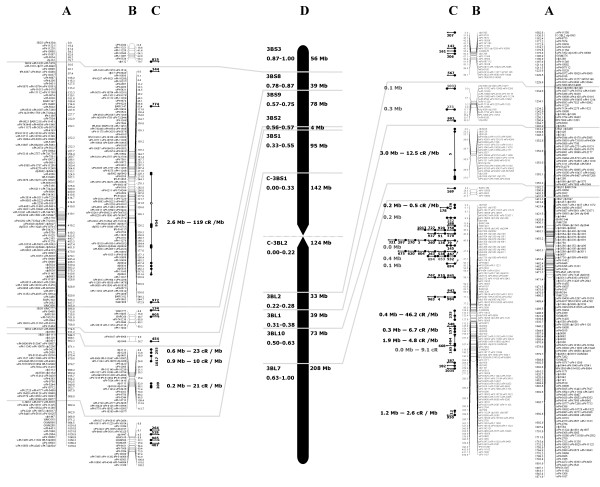
**Radiation hybrid map of wheat chromosome 3B.** (**A**) RH map of the short (left) and long (right) arm of chromosome 3B; map unit is centi-rays (cR). (**B**) RH map of the short (left) and long (right) arm of chromosome 3B divided into deletion bins. (**C**) BAC contig distribution based on anchored markers; contigs for which a breakage was identified are indicated by vertical arrows pointing in the resolved map orientation and cR/Mb resolution within the contig is shown; horizontal lines connecting multiple dots indicate contigs that had no breakage event and the size of the contig is reported; horizontal arrows indicate contigs for which only a single marker was mapped. (**D**) Deletion bins map of wheat chromosome 3B; no anchored markers were available for bin 3BL8-0.28-0.31 and 3BL9-0.38-0.50 so these were excluded from the reported bin map.

In plants, *in vivo* RH panels can be generated by simple gamma irradiation of seeds, followed by artificial cross-pollination of adult mutant lines. A RH panel for chromosome 3B was generated by gamma irradiating seeds of a normal durum line containing chromosome 3B (AABB, 2n = 2x = 28: 13” + 3B”) at 350 Gray (Gy) and crossing it to an aneuploid line that lacks this chromosome (13” + 3D”). A total of 184 RH_1_ lines were developed, and 92 RH_1_ lines were selected on the basis of genotyping data from 84 Insertion Site Based Polymorphism (ISBP, labeled cfp) marker loci [36]. The selected sub-population is composed of 70 lines with deletions of various sizes (retention frequency 0.500-0.999), nine lacked the entire chromosome (retention frequency < 0.020), and 13 retained the whole chromosome (retention frequency of 1.000) (Figure 1). The average retention frequency of this selected population was 0.89. Based on past experience, this small sample is a good representation of any larger RH population [34-36]. Locations of radiation-induced breaks on chromosome 3B were determined employing 541 markers, which include 96 ISBP, 19 Simple Sequence Repeats (SSRs; labeled barc or wmc) and 426 Diversity Array Technology (DArT; labeled wpt or tpt) markers. For 128 of these markers (115 PCR-based and 13 DArTs), the chromosomal location [38,39] and the bacterial artificial chromosome (BAC) contig of appurtenance were known (available at http://urgi.versailles.inra.fr/cgibin/gbrowse/wheat_ FPC_pub/). These markers were defined as ‘anchor markers’. The marker information was used to construct an iterative framework map of LOD 10 using a modified version of the Carthagene software package [40]; for details on the mapping algorithm see Additional file 1. The final map (3B-RH) spans 1871.9 cR, as defined by 202 unique loci (Figure 2). Assuming even distribution of markers along the chromosome, the overall marker density is one marker every 3.5 cR, or approximately 1.9 Mb based on the 993 Mb size of chromosome 3B [8]. Quality of the RH map was tested by comparison with two previously published genetic maps [36,41]. This examination revealed better conservation of marker loci order between the 3B-RH iterative map and Paux et al. [36] map than between the two genetic maps (Additional file 1 Figure S2). Further confirmation of the strength of this approach was provided by the marker wPt-0223 mapped on the 3B-RH map at position 414.8 cR, in between anchor markers cfp5042 and cfp5031, which are at positions 313.1 cR and 419.2 cR, respectively. Marker wPt-0223 was mapped into contig 954, which has been entirely sequenced [42]. The two anchor markers cfp5042 and cfp5031 are located at 1,103,689 bp and 1,640,531 bp on this contig, respectively. The sequence of wPt-0223 was used to map this locus *in silico* at position 2,170,833 bp (i.e. 0.53 Mb proximal of marker cfp5031), just outside the interval predicted in 3B-RH. Assuming 100% to be the error of placing a locus an entire chromosome length away (993 Mb) from its correct physical position, error for the 3B-RH map was calculated (0.53 Mb / 993 Mb) to be as low as 0.05%. This is a relatively small error considering that only 92 lines were used for the analysis.

### RH map resolution

Map resolution is defined as the minimum physical distance between two marker loci needed to map them separately. It is calculated as the ratio between physical and map distances (i.e. Mb divided by cR or cM). Smaller values indicate better resolution. Radiation hybrid maps are expected to provide higher map resolutions than genetic maps, and also a better estimate of the actual physical distance between mapped loci [43]. To verify these two hypotheses, the resolution was estimated at three physical levels: *i*) the whole chromosome (993 Mb), *ii*) chromosome sub-portions, defined as cytogenetic deletion bins (4 Mb to 208 Mb; Table 1), and *iii*) within BAC contigs (0.1 Mb to 3.0 Mb; Table 2). Cytogenetic deletion bins (hereafter referred to as ‘bins’) are physical segments of wheat chromosomes identified by cytogenetic lines carrying a terminal deletion of a specific chromosome fragment [44]. The physical sizes of these bins have been estimated through a combination of molecular and cytogenetic studies. These bins are commonly identified by the chromosome number (3B), the chromosome arm (S or L), and the percentage of the specific arm that is identified (i.e. 0.63-1.00, from 63% of the arm till the end, 100%); for simplicity, we report the bin full name in Table 1, and we only use an abbreviated identifier in the text. BAC contigs were generated by fingerprinting large libraries of clones, and their physical size is an estimate ([36]; Table 2). The resolution was calculated for both maps, genetic and RH, for the first two levels, but BAC contig analysis was reserved only for 3B-RH.

**Table 1:** Comparison of radiation hybrid and genetic maps of wheat chromosome 3B

**Deletion bins**	**3BS3-0.87-1.00**	**3BS8- 0.78-0.87**	**3BS9-0.57-0.75**	**3BS2-0.56-0.57**	**3BS1-0.33-0.55**	**C-3BS1-0.33**	**C-3BL2-0.22**	**3BL2-0.22-0.28**	**3BL1-0.31-0.50**	**3BL10-0.50-0.63**	**3BL7-0.63-1.00**	**Chr**^†^
No. of marker	7.0	155.0	9.0	3.0	44.0	28.0	27.0	29.0	47.0	4.0	187.0	540.0
Bin size (Mb)	56.0	39.0	78.0	4.0	95.0	142.0	124.0	33.0	39.0	73.0	208.0	992.0
Bin size (%)	5.7	3.9	7.9	0.4	9.6	14.3	12.5	3.3	3.9	7.4	21.0	89.8
Bin saturation												
(Mb Marker no^-1^)	8.0	0.3	8.7	1.3	2.2	5.1	4.6	1.1	0.8	18.3	1.1	1.8
Map size (cR)	79.7	572.9	69.2	36.7	113.7	115.6	99.1	58.9	37.0	41.3	459.6	1871.9
BR freq (cR 2 Mb^-1^) ^§^	0.7	7.4	0.5	4.6	0.6	0.4	0.4	0.9	0.5	0.3	1.1	1.0
Resolution (Mb cR^-1^) ^¶^	0.7	0.1	1.1	0.1	0.8	1.2	1.3	0.6	1.1	1.8	0.5	0.5
Map size (cM) ^‡^	5.2	33.1	13.4	--	1.6	0.9	1.6	1.6	6.2	3.5	104.2	179.0
CO freq. (cM Mb^-1^) ^§‡^	0.1	0.8	0.2	--	0.0	0.0	0.0	0.0	0.2	0.0	0.5	0.2
Resolution (Mb cM^-1^) ^¶^	10.8	1.2	5.8	--	61.3	167.1	80.0	21.3	6.3	21.2	2.0	5.5
Map size ratio (cR cM^-1^)	15.3	17.3	5.2	--	73.4	136.0	63.9	38.0	6.0	12.0	4.4	10.45

^†^ Whole chromosome values are not averages for each category but re-calculated for the overall values. ^‡^ Values in the row are as of Saintenac et al. [7]. ^§^ Frequency of DNA breakage and repair per Mb and frequency of a crossing-over event per Mb. ^¶^ Resolution is the potential to uniquely resolve two markers at a distance equal or larger than this value. *Chr*: chromosome; *BR freq*: breakage and repair frequency; *CO freq*: crossing over frequency; *cR*: centiRays; *Mb*: megabase.

**Table 2:** BAC contigs of known physical size mapped on radiation hybrid map of wheat chromosome 3B

**Contig ID**	**Size (Mb)**	**Size (cR)**	**Markers (count)**	**Resolution(Mb cR)**^**-1**^	**Deletion Bin**
Ctg0954	2.6	311.5	12	0.01	3BS8-0.78-0.87
Ctg0209	0.2	4.2	3	0.04	3BS1-0.33-0.55
Ctg0255	0.6	13.8	2	0.04	3BS1-0.33-0.55
Ctg1017	0.9	8.8	2	0.10	3BS1-0.33-0.55
Ctg0005	3.0	37.0	6	0.08	3BL1-0.31-0.38
Ctg0235	0.4	18.3	3	0.02	3BL7-0.63-1.00
Ctg0464	1.9	9.1	3	0.20	3BL7-0.63-1.00
Ctg0012	1.2	4.5	2	0.26	3BL7-0.63-1.00
Ctg0157	0.3	2.0	2	0.14	3BL7-0.63-1.00
Sub-total	10.9	418.3	35	^†^0.09	
Ctg0273	0.3	0.0	2		3BL2-0.22-0.28
Ctg1033	0.1	0.0	2		3BL2-0.22-0.28
Ctg0145	0.0	0.0	3		3BL7-0.63-1.00
Ctg0152	0.2	0.0	3		3BL7-0.63-1.00
Ctg0436	0.2	0.0	2		3BL7-0.63-1.00
Ctg0532	0.2	0.0	2		3BL7-0.63-1.00
Ctg0653	0.4	0.0	2		3BL7-0.63-1.00
Ctg0694	0.1	0.0	2		3BL7-0.63-1.00
Total	12.5		52		

The first nine contigs could be oriented, the remaining eight could not be oriented.

^†^ Average.

In 3B-RH map, the bin locations were known for 115 anchor markers, and this information was used to extrapolate the locations of the non-anchored markers. The largest portion of 3B-RH map is contained within two bins (3BS8 and 3BL7) accounting for 55% of the total map size, but only for 25% of the physical size of chromosome 3B (Table 1 and Figure 2). Assuming complete coverage of the chromosome, and considering the 3B physical size of 993 Mb [8], the 3B-RH map resolution averaged 0.53 Mb cR^-1^, ranging from 0.1 Mb cR^-1^ for bin 3BS8 and 3BS2 to 1.8 Mb cR^-1^ for 3BL10 (Table 1). The two centromeric bins exhibited very similar resolutions, 1.2 Mb cR^-1^ and 1.3 Mb cR^-1^ in C-3BS1 and C-3BL2, respectively (Table 1). Chromosome-wise, the RH map resolution deviated less than five-fold from the calculated average. In comparison, the resolution calculated by Saintenac et al. [7] for their genetic map of chromosome 3B was 5.5 Mb cM^-1^, 10.5 fold lower than the resolution of the 3B-RH map, and deviated along the chromosome up to 30 fold (1.2 and 167.1 Mb cM^-1^) from the average (Table 1).

The resolution of the 3B-RH map reached its minimum in bin 3BL10 at 1.8 Mb cR^-1^. Based on this value, the small population of 92 RH_1_ lines used should have the mapping potential to uniquely order any BAC contig with size >1.8 Mb. To test this hypothesis, markers anchored to 72 BAC contigs (Table 2 and Figure 2), 15 from the short arm and 57 from the long arm, mainly belonging to the bin 3BL7 (45 contigs), were analyzed. A contig can be assigned to a specific chromosomal position with the help of a single marker; however, two or more mapped markers are required to orient a contig. For 17 of 72 contigs two or more markers were available, and nine of these could be uniquely oriented (Table 2). The physical distance between the markers used to orient the contigs ranged from 3.0 Mb for contig 5, to 0.0 Mb (meaning that the markers are at a distance too small to be dissected by BAC fingerprinting but are not necessarily at the same locus, see [42]) for contig 145. As expected, the eight contigs that could not be oriented contained markers spaced by distances <1.8 Mb, ranging from 0.4 Mb to 0.0 Mb. Map resolution within contigs were also calculated, ranging from 0.01 Mb cR^-1^ for contig 954, to 0.26 Mb cR^-1^ for contig 12 (Table 2) and averaging 0.09 Mb cR^-1^ for the whole chromosome.

### Sizes of gamma ray induced chromosome deletions in the 3B-RH panel

A single radiation-induced deletion was defined by a set of flanking markers detecting an uninterrupted deletion smaller than a whole chromosome arm. Eight of the 92 lines had continuous deletions larger than a chromosome arm, retaining only a small portion of chromosome 3B, probably through translocation to a different chromosome. One line apparently lost the entire chromosome. The loss of an entire chromosome can be due to large rearrangements or improper chromosome sorting at meiosis, possibly caused by excessive deletions or removal of critical segments, such as the centromere. Thirteen putative RH lines did not show any deletions (Figure 1). These 22 lines were not considered when calculating the deletion size. In the absence of a fully sequenced genome, the deletion size was measured in map units (cR) and then converted into Mb, based on the conversion ratio calculated for each bin (i.e. resolution, Table 1).

**Figure 3:**
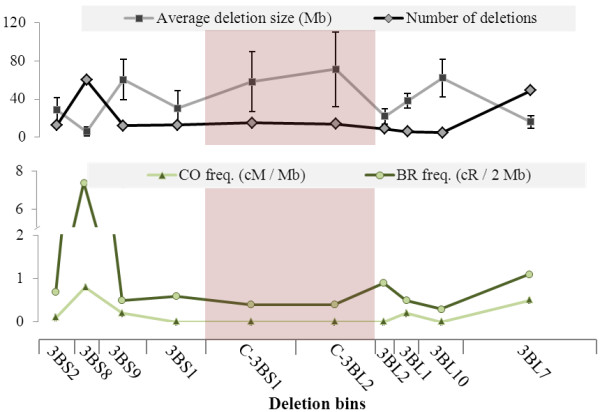
**Comparison of breakage/repair and crossing over frequencies, and deletion sizes across wheat chromosome 3B.** Positive and negative correlations between the average deletion size, the number of deletions, the frequency of crossing-over (CO) (as of Saintenac et al. [7]) and breakage/repair (BR) frequency across the 3B chromosome. For the average deletion size the standard deviation across lines is represented as error bars; the other values plotted are absolute with no replicates.

The largest deletion identified spans 120.2 cR, representing the loss of the entire bin C-3BS1 equal to 144 Mb, while the smallest deletion stretches 13.1 cR of bin 3BS8, accounting for 1.3 Mb in size. Deletions of smaller size are also possible [14] but their identification would require a more targeted approach than the one employed here. The average deletion size across chromosome 3B was 26.4 Mb, ranging from 5.9 Mb of bin 3BS8 to 71.4 Mb of bin C-3BL2 (Figure 3). On average, each of the 70 informative lines carried 2.8 deletions, ranging from one to thirteen (Figure 2). There is an inverse correlation (r = −0.64, p = 0.05) between the number of deletions and their relative size, with the centromeric regions typically having fewer but larger deletions and the telomeric regions having smaller and more frequent deletions.

**Figure 4:**
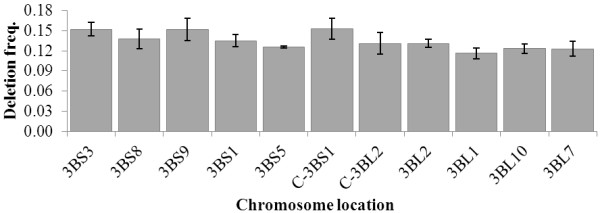
**Distribution of deletion frequencies across wheat chromosome 3B.** Each deletion bin is indicated by its designation (i.e. 3BS3) and represents a portion of chromosome 3B. The deletion frequency for each bin was calculated as the average of the deletion frequencies of each marker mapped within that bin. The error bars indicate the standard deviation of the values across the markers.

Also, the distribution of deletion frequency (defined as how frequently a given marker locus is lost in a population) across the RH-3B population was investigated in an attempt to identify any chromosomal region which is preferentially deleted or retained (Figure 4, Additional file 1 Figure S3). The average deletion frequency was 13%, reaching a maximum of 15% in C-3BS1 and a minimum of 12% in 3BL1. Overall the deletion frequency was evenly distributed and no significant difference was observed among the bins. Apparently, the vicinity to chromosomal landmarks such as the centromere or telomeres does not influence the frequency at which a given locus is lost, but rather the size or number of deletions that are created.

### Distribution of DNA break/repair events show correlation to the frequency of crossing-over

The cR unit measures the frequency of co-retention of two marker loci (i.e, one cR is one difference of the state (deleted *vs.* retained) between two adjacent loci observed in every 100 lines screened). Similarly, one cM indicates one recombination event between two loci in 100 lines. Deletion of one locus is likely the result of two DSBs, one distal and one proximal to the lost marker locus. Therefore, the number of deletions between two markers in 100 lines can be obtained by dividing the cR distance separating them by two. A DNA deletion in RH is the consequence of a radiation-mediated breakage, which is then repaired by the DNA-damage repair mechanism. Hence, when dividing half of the cR distance between two markers by the physical size that separates them, we are measuring the frequency at which radiation-mediated DSBs are formed and then repaired, most likely through NHEJ. This frequency (called break/repair (BR) frequency; Table 2) was calculated and expressed as the number of breakage/repair events (i.e. cR/2) per Mb. For instance, a BR frequency of 1 cR/2 Mb^-1^ would indicate that in a specific interval two breakage/repair events occurred on average every 1 Mb, while a value of 10 cR/2 Mb^-1^ would suggest 20 BR events every Mb. Similarly, by dividing cM by the physical size of the interval, we are measuring the average physical distance that separated crossing-over (CO frequency; [7]).

Crossing-over frequencies in wheat are known to be unevenly distributed across the chromosome, and generally decrease with proximity to the centromere [7]. The relative distance from the centromere does not entirely explain the distribution of CO events, still we confirmed a positive correlation (r = 0.604, p = 0.05) between the frequency of CO and the relative distance from the centromere. The same analysis did not return a significant interaction between the BR frequency and the distance from centromere (r = 0.292), further confirming that the proximity to chromosomal landmarks by itself does not influence the frequency of DNA breakage and repair. However, bin 3BS8 exhibited the highest BR frequency (7.4 cR/2 Mb^-1^, 7.7-fold higher than the chromosome average) and CO frequency (0.8 cM Mb^-1^, 4-fold higher than average). Bin 3BL10 exhibited one of the lowest CO frequency (0.05 cM Mb^-1^, 4-fold below average), and also the lowest BR frequency (0.3 cR/2 Mb^-1^, 3.6-fold lower than the chromosome average). The similarity between these values prompted us to compute chromosome-wide correlation between the BR frequencies and the CO frequencies (Figure 3). A correlation coefficient r = 0.879 (*p* = 0.01) was obtained when considering the ten bins for which information on both frequencies was available. Bin 3BS2 was excluded from the analysis due to lack of CO frequency data. This significant correlation indicates that the CO and BR frequencies are not independent values (Figure 3). Furthermore, the total number and average size of radiation-mediated deletions in each deletion bin was calculated, confirming that both CO and BR frequencies are positively correlated (*p* = 0.01) with the number of deletions, but only weakly inversely correlated with the average deletion size (*p* = 0.06) (Figure 3). No significant correlation was observed between CO, BR frequencies and markers deletion frequencies. To avoid confusion, we would like to emphasize that BR frequency and deletion frequency do not measure the same biological effect. The BR frequency value estimates the activity of the DNA repair mechanism, while the deletion frequency measures just the number of times that a given locus or a region is damaged by radiation.

## Discussion

In this study, a population of 92 RH_1_ lines was analyzed using a novel iterative framework mapping algorithm (Additional file 1) to generate a dense RH map of wheat chromosome 3B. The map quality tests indicated a small map error (0.05%). Hence, it was concluded that the method employed for mapping did not generate any perceivable bias, and downstream analyses should not be skewed by the anchor marker mapping approach.

Is RH mapping resolution higher and more uniform than the resolution of genetic mapping? To answer this question, the 3B-RH map was compared to the high quality genetic map of chromosome 3B published by Saintenac et al. [7]. The 3B-RH panel provided on average a 10.5 fold higher overall resolution than the genetic map, reaching a maximum of 136-fold better resolution at the centromere, where recombination is most scarce. We believe that this is sufficient evidence to conclude that RH mapping indeed provides higher resolution than genetic mapping. The average resolution is often calculated for genetic maps, but the uneven distribution of recombination along a chromosome can result in up to 30-fold variation between telomeric and centromeric regions (1.2 to 167.1 Mb cM^-1^; Table 1). For this reason, the resolution value calculated for genetic maps is not a reliable measure of the actual physical distance separating the mapped loci. To investigate if RH maps would provide a more uniform resolution across the chromosome, the average resolution for the entire chromosome was compared with the resolution for each chromosomal region. The average resolution, calculated as the total physical size of the chromosome divided by the total map length, was 0.53 Mb cR^-1^ and a maximum of five-fold deviation from this value (0.1 and 1.8 Mb cR^-1^) along the chromosome was observed. Thus RH mapping resolution is six times more uniform than genetic mapping resolution. The resolution within BAC contigs for this map was also assessed. In this case, resolution fluctuated more dramatically, ranging from 0.26 Mb cR^-1^ to 0.01 Mb cR^-1^ a 53-fold increase from the chromosome-wide average, and averaged at 0.09 Mb cR^-1^ (six-fold increase). This large fluctuation is probably the result of the non-random selection of RH lines identified at the beginning of the study. In fact, the subset of 92 lines was specifically selected for their quality of map-resolving 84 markers of the 109 employed to measure the BAC contig resolution. It is then not surprising that they provide a much higher resolution exactly for those markers that were used in the initial selection.

Overall, the lowest resolution observed for any chromosome region in this study was 1.8 Mb cR^-1^. This indicates the ability of our small RH population to unequivocally orient any BAC contig of size larger than 1.8 Mb. Only eight BAC contigs could not be uniquely oriented in this study, the largest of which was only 0.4 Mb in size. Such high resolution has been observed in many human and animal radiation hybrid maps before [21,27,29], but among plant studies reported to date only a resolution of ~0.2 Mb calculated on the number of obligate breaks for chromosome 1D of wheat [35] is close to the high resolution presented here.

### The chromatin state affects the DNA break/repair mechanism

RH studies rely on the random formation of deletions for mapping. Given the theoretical absence of molecular bias, the RH mapping approach has been thought to provide a true physical representation of chromosomes [13,35,45]. In contrast, genetic maps are not precise physical representation of the genome because they rely on recombination events that may not be evenly distributed [7,13,35,45]. The biased distribution of crossing-over events is associated with the specific requirements of the recombination machinery. In yeast and mouse, meiotic DSBs that initiate recombination are predominantly formed in open chromatin sites marked by trimethylation of lysine at position 4 in the H3 histone [46-48]. Little is known about patterns of meiotic DSB formation in plants [49], but they are likely similar to those in yeast and mammals, i.e. fewer DSBs are formed in the more densely packed chromatin regions [50]. Open chromatin regions should be more common in the distal regions of wheat chromosomes [7].

The data presented here confirms that the frequency of CO events are partially dependent on the relative distance from the centromere (*p* = 0.05) but fails to identify a similar correlation for BR frequency. This lack of correlation supports the hypothesis that RH maps are indeed true physical representations of chromosomes, and that the formation of deletions are random events independent of chromosome landmarks. However, it must be kept in mind that CO happens at meiosis, when chromosome are highly compacted, while BR occurs in somatic cells mostly during mitotic interphase, when chromosome landmarks are hard to observe. On the other hand, data presented here show dependent distribution between CO and BR events, suggesting a similar preference for specific chromosomal regions for both mechanisms. In literature, three plant studies have reported strong correlations between meiotic CO frequencies and somatic DNA break and repair processes. Liu et al. [51] showed that distribution of insertions of the *Mu* transposon in maize correlates with the distribution of recombination events across the genome. Similarly, Choulet et al. [42] identified correlation between transposable element (TE) distribution and disruption of gene order conservation, which is accelerated by COs. The third study demonstrated a correlation between TE distribution and modification of gene order, as well as a correlation between non-syntenic gene order and the CO frequency [52]. A common causative factor that explains the observed correlations is the dependence of all three processes, TE insertion, gene order disruption and CO formation, on formation of DSBs. Based on these reports and data presented here, one can postulate that the correlation between CO frequency and BR frequency can also be explained by the dependence on DSBs formation in both processes, and their consequent dependence on the chromatin state.

### A working hypothesis: how does the chromatin state affects the DNA-damage/repair mechanism?

Assuming that the breakage/repair mechanism has a preference for open chromatin regions, it is intriguing to hypothesize on how these regions specifically influence this process. There are two levels at which the state of chromatin could influence formation of chromosomal deletions: *i*) regions of compact chromatin could be more resistant to radiation damage; and/or *ii*) the repair mechanism requires open chromatin regions to perform its activity. If the first hypothesis is correct, we should observe a reduction in the number of deletions in those regions that are particularly heterochromatic, such as the peri- and centromeric regions. The data presented in Figure 4 (and extended in Additional file 1 Figure S3 in SI) show no significant difference in the frequency at which deletions forms in the telomere or centromere of chromosome 3B. This would suggest that chromatin compactness by itself is not a sufficient shield to prevent DNA damage. A similar conclusion was also reached for yeast [53] and humans [54], leaving our first hypothesis short of supporting evidences. On the other hand, it leaves open the possibility that the chromatin state directly influences the repair mechanism, possibly preventing the formation of non-radiation-mediated DSBs, necessary to complete the repair. Goodarzi et al. [55] demonstrated that in human cells the repair complex is unable to adequately access or manipulate radiation-mediated breaks occurring in regions of compact chromatin. Hence, if radiation-mediated breaks happen independently from the chromatin state, but less condensed chromatin is necessary for the DNA repair mechanism to properly operate, logic dictates that in tightly compacted chromosomal regions larger and less frequent deletions are expected, while in more open regions the number of deletions would increase and their size diminishes. That is precisely what we observed for chromosome 3B (Figure 3). Thus this data supports *in vivo* the hypothesis that “higher order chromatin architecture exerts just as profound an influence on DNA repair as it does on nuclear processes such as transcription and replication” [54].

## Conclusion

Radiation hybrid mapping is an effective approach to map all markers (monomorphic or otherwise) in wheat and other organisms. High levels of mapping resolution can be achieved with relatively small populations. Since its first application 37 years ago [12] RH mapping was believed an approach totally independent from patterns of recombination. Here, for the first time, data have been collected that suggest otherwise, indicating that RH mapping relies on higher order chromatin structure similar to recombination hot-spots observed in genetic mapping. However, the 3B-RH map generated still offered a resolution eleven fold higher than a comparable genetic map and a fairly consistent physical to cR conversion across the entire chromosome, making this approach the most dependable for the scaffold assembly of genome sequencing initiatives. Moreover, in plants RH lines can be produced entirely *in vivo*, providing a unique tool to study the effect of radiation in living organisms. New insights have been gathered in the past few years on possible interactions between the state of chromatin and various DNA break-repair processes, such as those involved in CO event formation, DNA repair, TE insertion, and synteny disruption. We presented here a working hypothesis to explain how the chromatin state could affect the DNA repair mechanism, together with the biological material to further investigate this hypothesis. Specifically targeted cytogenetic studies employing RH lines are likely to provide the evidences necessary to shed light on the precise effect of chromatin on the DNA break-repair mechanism.

## Methods

### RH mapping population

The 3B-RH panel was generated as described in Paux et al… [36] by crossing the durum wheat *Triticum durum* L. var. ‘Langdon’ (LDN) after irradiation at 350 Gy of gamma ray to the aneuploid ‘Langdon’ 3D(3B) (LDN 3D(3B)). RH_1_ seeds were planted under controlled greenhouse conditions and DNA was extracted from leaves of four weeks old plants as described earlier [34]. Non-irradiated double monosomic (13” + 3B’ + 3D’) F_1_ lines were also generated and employed as experimental controls. All DNA samples were equilibrated to concentrations of 50 ng per μl. A total of 184 RH_1_ lines were initially employed in this study, but only 92 selected lines were fully genotyped.

### Molecular analysis

Genotyping was conducted with three classes of markers: cfp are PCR-based ISBP [56,57]; barc, gwm, and gpw are PCR-based SSR (http://wheat.pw.usda.gov/GG2/index.shtml); wPt and tPt are DArT markers (Canberra, AU) [41]. To guarantee chromosome 3B specificity, all markers were tested for positive amplification (ISBP and SSR) or hybridization (DArT) of the double monosomic F_1_ control line, and no-amplification / hybridization of LDN 3D(3B). In order to distinguish PCR failure from deletion-detection, all the markers amplifying a single 3B specific band were multiplexed with the control marker DEASY (Duplexing EASY) amplifying 164 bp of a chloroplastic ATP syntase alpha subunit [GebBank:M16842]. All PCR protocols have been described previously [36,39,56,57]. Ninety-two samples plus four experimental controls were genotyped in duplicate using the 3B specific DArT array following the protocol described in Wenzl et al. [41]. The deletion frequency is calculated as the number of loci with deletion divided the total number of loci genotyped, while the retention frequency is one minus the deletion frequency. The 3B genetic map resolution and CO frequency was calculated in Saintenac et al. [7]. The Carthagene mapping software v1.2.2 [40] was modified and used to generate a RH map of the entire 3B chromosome. Details on the superimposed modifications are available in Additional file 1.

### Statistical analysis

All correlation analyses were performed using the SAS 9.3 environment (SAS Institute, Cary, NC) and the correlation significance was determined on the basis of the Pearson product–moment correlation coefficient for a two-tail test with N-2 degrees of freedom, where N is the number of deletion bins considered [58].

## Abbreviations

RH: Radiation hybrid; DSB: Double strand break; HR: Homology-directed repair; NHEJ: Non-homologous end-joining; BAC: Bacterial artificial chromosome; cR: centi Rays; Gy: Gray; ISBP: Insertion site based polymorphism; SSR: Simple sequence repeat; DArT: Diversity Array Technology; BR: Break/repair; CO: Crossing over; LDN: ‘Langdon’; LDN 3D(3B): ‘Langdon’ with chromosome 3B substituted by chromosome 3D; DEASY: Duplexing EASY.

## Competing interests

The authors declare that they have no competing interests.

## Authors’ contributions

A.Kumar, F.M.B., E.P., M.M. de J., Y.Q.G., C.F., and S.F.K designed research; A.Kumar, F.M.B., M.M. de J. S.K., A.G., V.T. and M.D. performed the research; E.P., E.H., A.Kilian, and C.F. provided access to advanced analytical tools and unpublished data; O.Al-A. and A.M.D. developed iterative framework mapping script; H.S.B., H.S.D., P.K.G., and G.S.R. provided support for student interns A.Kumar, S.K., A.G., and V.T.; F.M.B., A. Kumar, E.P., M.M. de J., W.P.P and E.H. analyzed the data; F.M.B, A.Kumar, E.P.,W.P.P., S.F.K. wrote the paper, M.J.I. and S.F.K. managed the project. All authors read and approved the final manuscript.

## Supplementary Material

Additional file 1 **Supplementary text, tables and figures.** The file contains supplementary text, Table S1, Figure S1, S2 and S3. Suppl. Text describes the rational of radiation hybrid mapping, the algorithm developed to exploit the specific characteristics of this type of mapping, and its proof of concept. Table S1 presents the statistical details of the iterative frame work mapping approach applied to the radiation hybrid map of chromosome 3B. Figure S1 shows the superior marker order conservation between 3B-radiation hybrid (3B-RH) map and the 3B genetic map when employing iterative frame work mapping algorithm, instead of a non-iterative approach. Figure S2 shows how the error in marker order conservation between the 3B-RH map and the 3B genetic map is lower than the error that exists between published genetic maps. Figure S3 shows that marker loci have non significantly different deletion frequencies throughout the 3B chromosome [36,41,59]. Click here for file
