# Supplementary material for: DNA repair and crossing over favor similar chromosome regions as discovered in radiation hybrid of Triticum
Source: BMC Genomics. 2012 Jul 24;13:339. doi: 10.1186/1471-2164-13-339 (PMC3443642; doi:10.1186/1471-2164-13-339)
Supplement: Additional file 1 — Supplementary text, tables and figures. The file contains supplementary text, Table S1, Figure S1, S2 and S3. Suppl. Text describes the rational of radiation hybrid mapping, the algorithm developed to exploit the specific characteristics of this type of mapping, and its proof of concept. Table S1 presents the statistical details of the iterative frame work mapping approach applied to the radiation hybrid map of chromosome 3B. Figure S1 shows the superior marker order conservation between 3B-radiation hybrid (3B-RH) map and the 3B genetic map when employing iterative frame work mapping algorithm, instead of a non-iterative approach. Figure S2 shows how the error in marker order conservation between the 3B-RH map and the 3B genetic map is lower than the error that exists between published genetic maps. Figure S3 shows that marker loci have non significantly different deletion frequencies throughout the 3B chromosome [36,41,59]. [file 1471-2164-13-339-S1.docx]

**Supplementary Information**

**Supplementary text: Iterative framework map imposed on Carthagene**

The RH population used in this study exhibited an average deletion size of ~10 Mb, with generally three deletions per line. Assuming perfect distribution of deletions across the entire 993 Mb of chromosome 3B, a minimum of 50 RH lines (50 × 3 deletions × 6.6 Mb ~ 993 Mb) would be required to cover the entire physical size of the chromosome. Assuming non-perfect conditions, where each deletion is not unique, larger population possibly double this size or more would be required to generate a comprehensive RH map. The very advantage of using small deletions to obtain high level of map resolution is also the main drawback of RH mapping. Genetic mapping relies on large recombination blocks to order marker loci. Thus markers physically distant on the chromosome can show linkage and be correctly mapped. RH mapping instead employs very small ‘deletion blocks’ (~10 Mb in size) to link markers. Hence, two markers that are 100 Mb apart can possibly be connected by one recombination block (i.e. one recombinant line) but would require at least 10 deletion blocks.

To overcome this limitation, we gathered or produced physical information for 115 of the genotyped markers (anchor markers). Initially, we created a framework map for the 115 anchor markers. This map was generated using Carthagene “build”, “annealing”, “flips”, and “polish” functions and then hand curated to assure that all the biological evidence were respected, such as bin-location or contig assignment. The remaining 426 markers were merged on the framework map using the “buildfw” function. This was achieved through iterative analysis. Markers were assigned to any interval between two anchor markers using the command “buildfw” with LOD score of 10. These assigned markers, plus the two initial inner anchor markers, and two outer anchor markers of the framework map, were used for mapping in Carthagene using the commands “build-10”, “annealing”, “flips”, and “polish”. All the markers mapping in between the two anchor markers underlying a specific interval were merged into the new framework map; all other markers were discarded and reused in the following iteration. For instance if 15 markers were assigned the location between markers b and c these 15 markers and markers b and c, along with marker b’s outer neighbor (assume marker name is a) and marker c’s outer neighbor (assume marker name is d) are mapped. We only accept the list of marker orders that satisfy the condition: a, b, <markers list>, c, d. any other marker that does not satisfy this condition will be through out to be used in the next iteration. The new framework map, containing both the anchor markers and the most associated non-anchor markers, was used in a second iteration to merge the markers not yet incorporated. A total of ten iterations were necessary to map all the 426 non-anchored markers (Table S1). To determine the quality of this approach, it was initially compared to normal RH mapping using Carthagene “build”, “annealing”, “flips”, and “polish” functions, without iterative framework mapping. The on-line version of AutoGraph [59; <http://autograph.genouest.org/>] was used to graphically compare the two RH maps generated with and without iterative framework mapping to a good quality genetic map available in the literature (Fig. S1). Since the iterative approach showed better marker order conservation with the genetic map, we consider iterative framework mapping a good strategy for RH genotyping data.

**Table S1 Details of single iteration for iterative Carthagene frame-work mapping algorithm to produce the 3B-RH map**

| Iteration | Mapped markers | Markers left | Map quality | Total Map Size |
| --- | --- | --- | --- | --- |
| --------------------------- No. ---------------------------- | | | -- log10-likelihood -- | ------- cR ------- |
| 0 | 128 | 413 | -379.2 | 1010.7 |
| 1 | 219 | 322 | -506.86 | 1322.7 |
| 2 | 292 | 249 | -568.58 | 1466.6 |
| 3 | 312 | 229 | -585.12 | 1501.9 |
| 4 | 380 | 161 | -609.09 | 1568.3 |
| 5 | 430 | 111 | -629.04 | 1612.6 |
| 6 | 470 | 71 | -641.95 | 1650.3 |
| 7 | 503 | 38 | -668.01 | 1713.5 |
| 8 | 520 | 21 | -678.26 | 1730.3 |
| 9 | 528 | 13 | -699.81 | 1800.3 |
| 10 | 541 | 0 | -727.23 | 1871.9 |
|  |  |  |  |  |

**
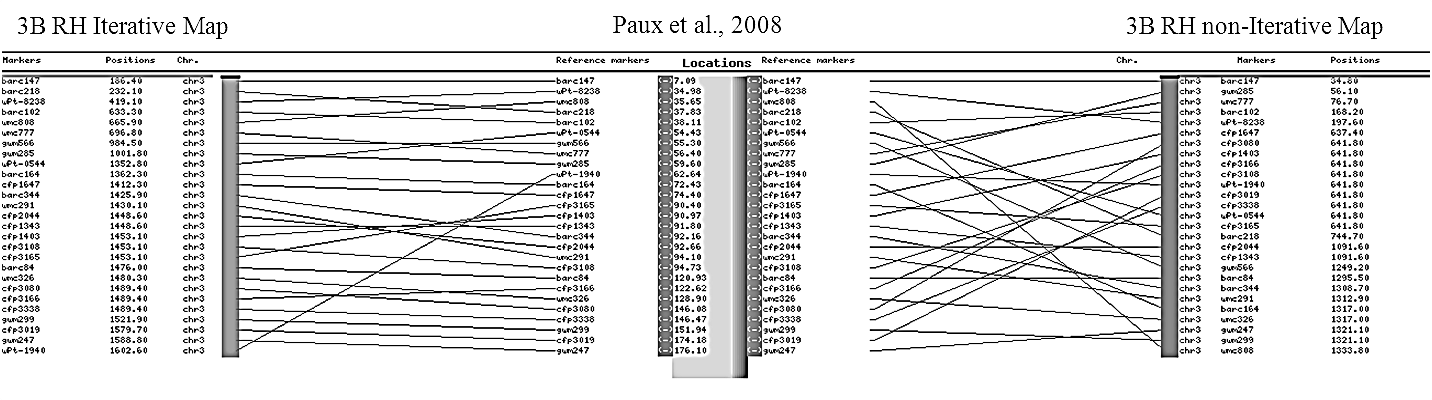
Figure S1AutoGRAPH output comparing one genetic map [36] to two RH maps** produced from the same dataset but applying two different mapping algorithms, the newly developed Iterative-Framework Map and the classical Carthagene. Good markers order conservation is indicated by parallel lines.

**
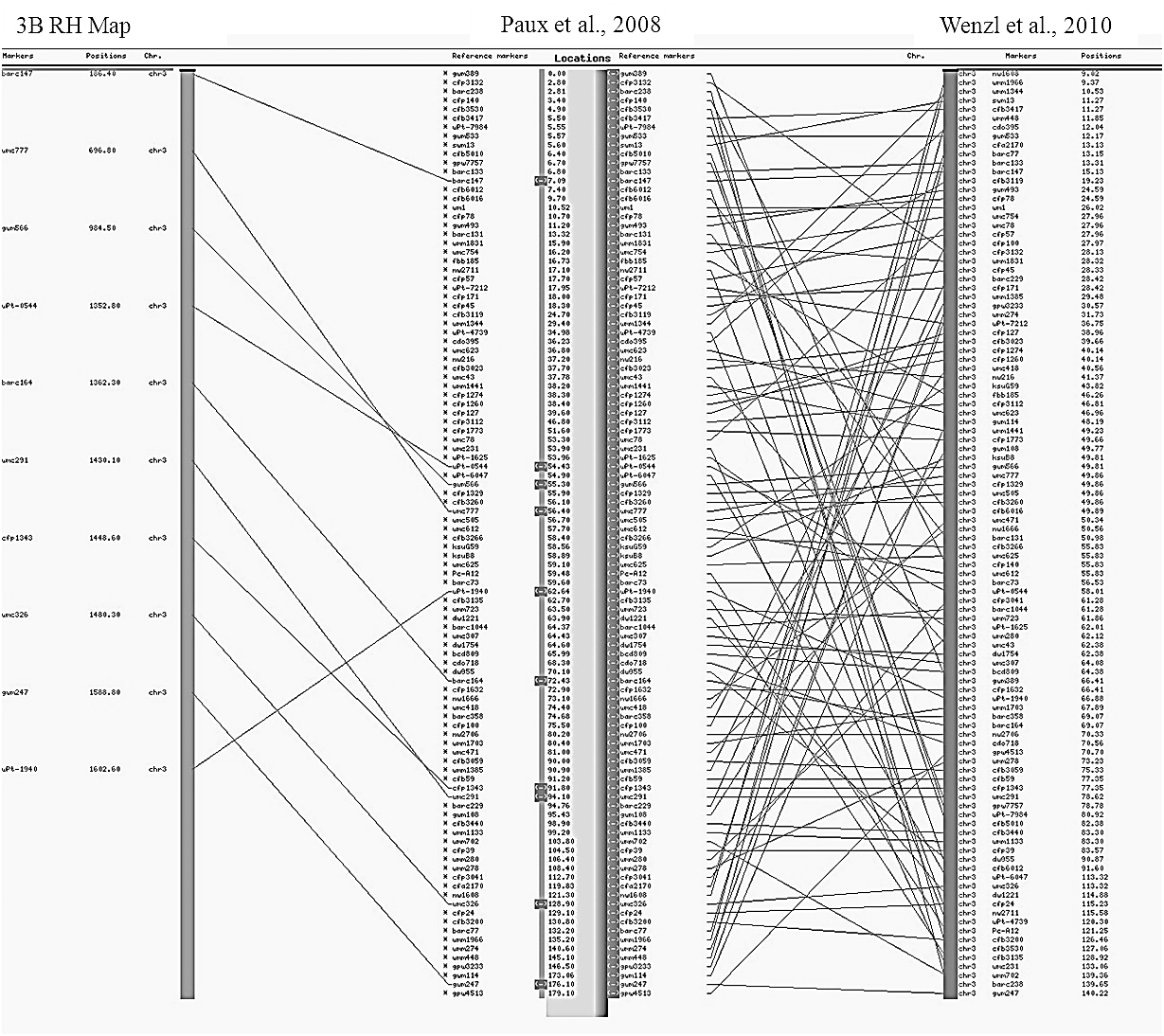
**

**Figure S2AutoGRAPH output comparing two genetic maps [36,41] to 3B-RH**. Good markers order conservation is indicated by parallel lines.

**
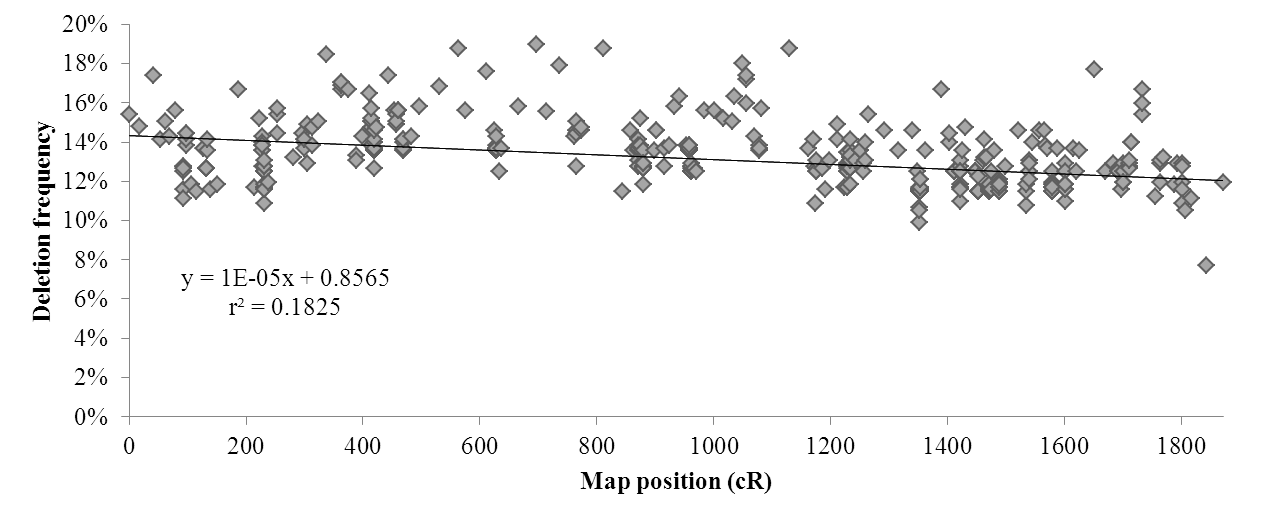
**

**Figure S3Deletion frequency distribution throughout the 3B-RH map considering all of the 541 markers**. Data points are represented in their mapping position along chromosome 3B.

**References**

36. [Paux E](http://www.ncbi.nlm.nih.gov/pubmed?term=%22Paux%20E%22%5BAuthor%5D), [Sourdille P](http://www.ncbi.nlm.nih.gov/pubmed?term=%22Sourdille%20P%22%5BAuthor%5D), [Salse J](http://www.ncbi.nlm.nih.gov/pubmed?term=%22Salse%20J%22%5BAuthor%5D), [Saintenac C](http://www.ncbi.nlm.nih.gov/pubmed?term=%22Saintenac%20C%22%5BAuthor%5D), [Choulet F](http://www.ncbi.nlm.nih.gov/pubmed?term=%22Choulet%20F%22%5BAuthor%5D), [Leroy P](http://www.ncbi.nlm.nih.gov/pubmed?term=%22Leroy%20P%22%5BAuthor%5D), [Korol A](http://www.ncbi.nlm.nih.gov/pubmed?term=%22Korol%20A%22%5BAuthor%5D), [Michalak M](http://www.ncbi.nlm.nih.gov/pubmed?term=%22Michalak%20M%22%5BAuthor%5D), [Kianian S](http://www.ncbi.nlm.nih.gov/pubmed?term=%22Kianian%20S%22%5BAuthor%5D), [Spielmeyer W](http://www.ncbi.nlm.nih.gov/pubmed?term=%22Spielmeyer%20W%22%5BAuthor%5D), [Lagudah E](http://www.ncbi.nlm.nih.gov/pubmed?term=%22Lagudah%20E%22%5BAuthor%5D), [Somers D](http://www.ncbi.nlm.nih.gov/pubmed?term=%22Somers%20D%22%5BAuthor%5D), [Kilian A](http://www.ncbi.nlm.nih.gov/pubmed?term=%22Kilian%20A%22%5BAuthor%5D), [Alaux M](http://www.ncbi.nlm.nih.gov/pubmed?term=%22Alaux%20M%22%5BAuthor%5D), [Vautrin S](http://www.ncbi.nlm.nih.gov/pubmed?term=%22Vautrin%20S%22%5BAuthor%5D), [Bergès H](http://www.ncbi.nlm.nih.gov/pubmed?term=%22Berg%C3%A8s%20H%22%5BAuthor%5D), [Eversole K](http://www.ncbi.nlm.nih.gov/pubmed?term=%22Eversole%20K%22%5BAuthor%5D), [Appels R](http://www.ncbi.nlm.nih.gov/pubmed?term=%22Appels%20R%22%5BAuthor%5D), [Safar J](http://www.ncbi.nlm.nih.gov/pubmed?term=%22Safar%20J%22%5BAuthor%5D), [Simkova H](http://www.ncbi.nlm.nih.gov/pubmed?term=%22Simkova%20H%22%5BAuthor%5D), [Dolezel J](http://www.ncbi.nlm.nih.gov/pubmed?term=%22Dolezel%20J%22%5BAuthor%5D), [Bernard M](http://www.ncbi.nlm.nih.gov/pubmed?term=%22Bernard%20M%22%5BAuthor%5D), [Feuillet C](http://www.ncbi.nlm.nih.gov/pubmed?term=%22Feuillet%20C%22%5BAuthor%5D): **A physical map of the 1-gigabase bread wheat chromosome 3B.***Science* 2008, **322:** 101-104.

41. [Wenzl P](http://www.ncbi.nlm.nih.gov/pubmed?term=%22Wenzl%20P%22%5BAuthor%5D), [Suchánková P](http://www.ncbi.nlm.nih.gov/pubmed?term=%22Such%C3%A1nkov%C3%A1%20P%22%5BAuthor%5D), [Carling J](http://www.ncbi.nlm.nih.gov/pubmed?term=%22Carling%20J%22%5BAuthor%5D), [Simková H](http://www.ncbi.nlm.nih.gov/pubmed?term=%22Simkov%C3%A1%20H%22%5BAuthor%5D), [Huttner E](http://www.ncbi.nlm.nih.gov/pubmed?term=%22Huttner%20E%22%5BAuthor%5D), [Kubaláková M](http://www.ncbi.nlm.nih.gov/pubmed?term=%22Kubal%C3%A1kov%C3%A1%20M%22%5BAuthor%5D), [Sourdille P](http://www.ncbi.nlm.nih.gov/pubmed?term=%22Sourdille%20P%22%5BAuthor%5D), [Paul E](http://www.ncbi.nlm.nih.gov/pubmed?term=%22Paul%20E%22%5BAuthor%5D), [Feuillet C](http://www.ncbi.nlm.nih.gov/pubmed?term=%22Feuillet%20C%22%5BAuthor%5D), [Kilian A](http://www.ncbi.nlm.nih.gov/pubmed?term=%22Kilian%20A%22%5BAuthor%5D), [Dolezel J](http://www.ncbi.nlm.nih.gov/pubmed?term=%22Dolezel%20J%22%5BAuthor%5D): **Isolated chromosomes as a new and efficient source of DArT markers for the saturation of genetic maps.***TheorAppl Genet* 2010, **121:** 465-474.

59. Derrien T, Andre C, Galibert F, Hitte C: AutoGRAPH: **an interactive web server for automating and visualizing comparative genome maps.***Bioinformatics* 2007, **23**:498-499.
